# Development and Evaluation of Small Peptidomimetic Ligands to Protease-Activated Receptor-2 (PAR_2_) through the Use of Lipid Tethering

**DOI:** 10.1371/journal.pone.0099140

**Published:** 2014-06-13

**Authors:** Scott Boitano, Justin Hoffman, Dipti V. Tillu, Marina N. Asiedu, Zhenyu Zhang, Cara L. Sherwood, Yan Wang, Xinzhong Dong, Theodore J. Price, Josef Vagner

**Affiliations:** 1 Arizona Respiratory Center and Department of Physiology, University of Arizona, Tucson, Arizona, United States of America; 2 The BIO5 Collaborative Research Institute, University of Arizona, Tucson, Arizona, United States of America; 3 Department of Pharmacology, University of Arizona, Tucson, Arizona, United States of America; 4 State Key Laboratory of Oral Diseases, West China Hospital of Stomatology, Sichuan University, Chengdu, China; 5 Department of Neuroscience, Johns Hopkins University, Baltimore, Maryland, United States of America; Medical School of Hannover, Germany

## Abstract

Protease-activated receptor-2 (PAR_2_) is a G-Protein Coupled Receptor (GPCR) activated by proteolytic cleavage to expose an attached, tethered ligand (SLIGRL). We evaluated the ability for lipid-tethered-peptidomimetics to activate PAR_2_ with *in vitro* physiological and Ca^2+^ signaling assays to determine minimal components necessary for potent, specific and full PAR_2_ activation. A known PAR_2_ activating compound containing a hexadecyl (*Hdc*) lipid via three polyethylene glycol (*PEG*) linkers (2at-LIGRL-*PEG*_3_-*Hdc*) provided a potent agonist starting point (physiological EC_50_ = 1.4 nM; 95% CI: 1.2–2.3 nM). In a set of truncated analogs, 2at-LIGR-*PEG*_3_-*Hdc* retained potency (EC_50_ = 2.1 nM; 1.3–3.4 nM) with improved selectivity for PAR_2_ over Mas1 related G-protein coupled receptor type C11, a GPCR that can be activated by the PAR_2_ peptide agonist, SLIGRL-NH_2_. 2at-LIG-*PEG*_3_-*Hdc* was the smallest full PAR_2_ agonist, albeit with a reduced EC_50_ (46 nM; 20–100 nM). 2at-LI-*PEG*_3_-*Hdc* retained specific activity for PAR_2_ with reduced EC_50_ (310 nM; 260–360 nM) but displayed partial PAR_2_ activation in both physiological and Ca^2+^ signaling assays. Further truncation (2at-L-*PEG*_3_-*Hdc* and 2at-*PEG*_3_-*Hdc*) eliminated *in vitro* activity. When used *in vivo*, full and partial PAR_2_ *in vitro* agonists evoked mechanical hypersensitivity at a 15 pmole dose while 2at-L-*PEG*_3_-*Hdc* lacked efficacy. Minimum peptidomimetic PAR_2_ agonists were developed with known heterocycle substitutes for Ser_1_ (isoxazole or aminothiazoyl) and cyclohexylalanine (Cha) as a substitute for Leu_2_. Both heterocycle-tetrapeptide and heterocycle-dipeptides displayed PAR_2_ specificity, however, only the heterocycle-tetrapeptides displayed full PAR_2_ agonism. Using the lipid-tethered-peptidomimetic approach we have developed novel structure activity relationships for PAR_2_ that allows for selective probing of PAR_2_ function across a broad range of physiological systems.

## Introduction

Protease-activated receptors (PARs) are a sub-family of G-protein coupled receptors (GPCRs) that have a unique mode of activation. PARs contain an embedded ligand that is exposed following proteolytic cleavage of the extracellular oriented NH_2_ terminus [1]. The different N-termini of the PARs present substrates for a variety of proteases that create selective activation (or inactivation) mechanisms for signal transduction [2], [3], [4]. The most common, diffusionally limited “tethered ligand” uncovered following trypsin-like serine protease activity of PAR_2_ [exposing SLIGKV (human) or SLIGRL (rodent)] serves as a potent agonist to the receptor. As an obvious consequence of its activation mechanism, PAR_2_ is associated with pathologies that have a strong protease release, including inflammatory related diseases such as arthritis, asthma, inflammatory bowel disease, sepsis, and pain disorders [1], [2], [4]. Stimulation of PAR_2_ in pain-sensing primary sensory neurons (nociceptors) leads to the sensitization of a variety of receptors including the noxious heat and capsaicin receptor TRPV1 [5], [6], [7]. This sensitization of sensory neuronal channels underlies thermal [7], [8], [9] or mechanical hypersensitivity [8], [10], [11] elicited by activation of PAR_2_. The involvement of PAR_2_ in pain and other pathologies makes it a prime target for drug discovery. Importantly, PAR_2_ has been associated with itch based partly on data obtained using the relatively potent PAR_2_ signaling peptide, SLIGRL-NH_2_. It is now clear that this peptide also stimulates an additional GPCR, Mas1 related G-protein coupled receptor type C11 (MrgprC11), and this receptor is responsible for the pruritic properties of SLIGRL-NH_2_ [12]. Therefore, assessing the selectivity of PAR_2_ ligands against receptors that are selectively expressed in sensory ganglia (e.g., MrgprC11; [13], [14]) is critical to developing selective probes for PAR_2_.

Small peptides or peptidomimetics that mimic the ligand binding properties of the tethered ligand exposed by proteolysis of the N-terminus of the receptor have been used to directly activate PARs [2], [15], [16], [17]. Activating peptides (e.g., SLIGKV-NH_2_ and SLIGRL-NH_2_) and peptidomimetics (e.g., 2-furoyl-LIGRLO-NH_2_ [18] and 2at-LIGRL-NH_2_ [19]) have provided useful tools for establishment of structure-activity relationships (SAR) and rational drug design because they limit off-target effects that are often a complication of natural protease activation. Early SAR studies suggested that the minimal peptide sequence required for PAR_2_ activation is a pentamer (either SLIGR-NH_2_ or the less potent LIGRL-NH_2_ [17], [20]). More recently, heterocycle-dipeptide mimetics have been shown to retain PAR_2_ activity [21]. However, full characterization of these shortened compounds has been hindered by a lack of assays sufficiently sensitive to evaluate full concentration responses. Commonly used assays require high concentrations (> 50 µM) that potentially limit PAR_2_-selectivity or prevent full solubility for preferred Ca^2+^ activation studies [21]. It is now evident that a variety of GPCRs, including PAR_2_, can elicit signaling pathway-specific activation with distinct physiological responses [4], [22], [23], [24], [25], [26]. A means to establish better evaluation of the minimal peptidomimetic structure required for full PAR_2_ activation would benefit PAR_2_ ligand discovery efforts.

Lipidation of peptide receptor agonists has been used to increase their potency via a variety of mechanisms [27]. Because of the naturally tethered ligands in PAR_2_, we hypothesized that lipidation of peptide and peptidomimetic agonists could provide a membrane bound tether to better mimic the natural receptor activation and thus increase their potencies [28]. Modification of the potent PAR_2_ peptidomimetic agonists 2at-LIGRL-NH_2_ and 2at-LIGRLO-NH_2_ with polyethylene glycol (*PEG*) spacers and a hexadecyl (*Hdc*) or a palmitoyl (*Pam*) group (e.g., 2at-LIGRL-*PEG*_3_-*Hdc* or 2at-LIGRLO(*PEG*_3_-*Pam*)-NH_2_) improves ligand potency to the low nanomolar range without sacrificing specificity to PAR_2_ as demonstrated in cell lines or in cells isolated from PAR_2_ wild type vs. PAR_2_^-/-^ mice [28]. Because of this increased potency, we hypothesized that this synthetic tethered ligand (STL) approach could be used to more closely examine SAR of peptidomimetics in an effort to better understand the minimal components necessary to specifically activate PAR_2_. In this report, we used the STL approach coupled with real time cell analysis (RTCA) and digital Ca^2+^ imaging microscopy to evaluate 14 compounds. We describe six STL compounds consisting of full or truncated parent peptidomimetic (2at-LIGRL-NH_2_) linked to three *PEG*s and one *Hdc* and evaluate their potencies, efficacies and specificities at PAR_2_, including screening against MrgprC11, to determine a minimal sequence necessary for specific activation of PAR_2_ *in vitro* and *in vivo*. Moreover, we used a parallel approach to fully evaluate potency of heterocycle di- and tetra-peptide mimetics using Ser_1_ and Leu_2_ substitutions known to activate PAR_2_ [19], [21]. These findings identify a minimal structure required for specific full and/or partial activation of PAR_2_ and thus, further elucidate highly potent and specific probes to examine the function of this receptor *in vitro* and *in vivo*.

## Materials and Methods

### Synthesis Materials

N^α^-9H-fluoren-9-ylmethoxycarbonyl (N^α^-Fmoc) protected amino acids, 2-(1H-benzotriazol-1-yl)-1,1,3,3-tetramethyluronium hexafluoro-phosphate (HBTU), and N-hydroxybenzotriazole (HOBt) were purchased from SynPep (Dublin, CA) or from Novabiochem (San Diego, CA). Aldehyde (4-(4-formyl-3-methoxyphenoxy)butyrylaminomethyl) resin and Rink Amide resins were acquired from Novabiochem (San Diego, CA). N,N’-diisopropylcarbodiimide (DIC) and diisopropylethylamine (DIEA) were purchased from IRIS Biotech (Marktredwitz, Germany). A *N^g^*-2,2,4,6,7-pentamethyl-dihydrobenzofuran-5-sulfonyl side chain protecting group was used for Arg. 2-aminothiazole-4-carboxylic acid and 5-isoxazole-carboxylic acid were obtained from Combi-Blocks (San Diego, CA); hexadecyl (*Hdc*-NH_2_) amine was obtained from Sigma-Aldrich. Fmoc-protected version of *PEG* (1-(9H-fluoren-9-yl)-3,19-dioxo-2,8,11,14,21-pentaoxa-4,18-diazatricosan-23-oic acid) was obtained from Novabiochem (San Diego, CA). Reagent grade solvents, reagents, and acetonitrile for High Performance Liquid Chromatography (HPLC) were acquired from VWR (West Chester, PA) or Aldrich-Sigma (Milwaukee, WI), and were used without further purification unless otherwise noted. Compounds were manually assembled using 5 mL plastic syringe reactors equipped with a frit, and a Domino manual synthesizer obtained from Torviq (Niles, MI). The C-18 Sep-Pak™ Vac RC cartridges for solid phase extraction were purchased from Waters (Milford, MA).

### Compound Synthesis

**Figure 1:**
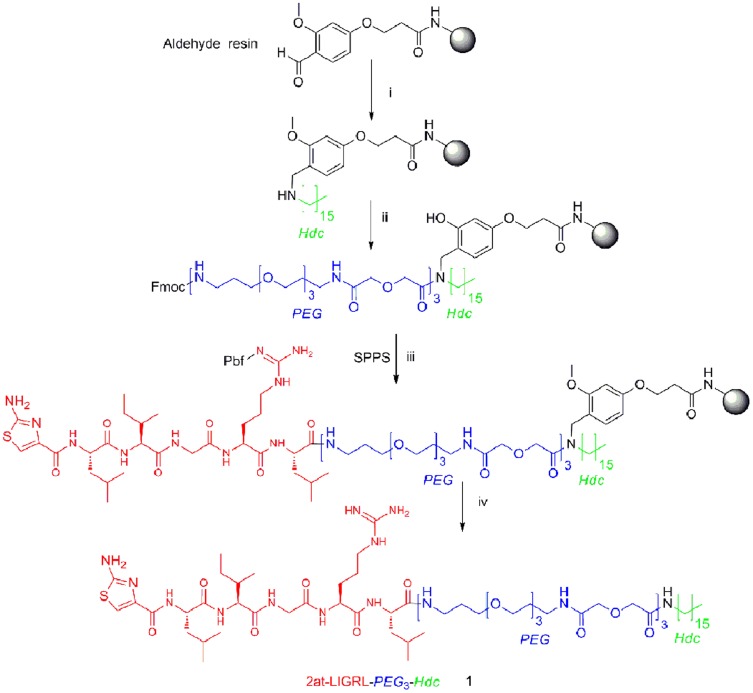
Synthetic route for 2at-LIGRL-*PEG*_3_-*Hdc* (compound 1). *i* Reductive alkylation: hexadecyl amine (5 equivalents), sodium cyanoborohydride (5 equivalents) in 5% acetic acid in DCM (0.25 M solution), overnight; ***ii*** a) Fmoc-*PEG*(6 equiv), DIC (3 equivalents) for first coupling b) Piperidine/DMF (1∶9) for Fmoc deprotection ***iii*** Fmoc/tBu synthesis continued as follows: a) Fmoc-aa-OH (3 equivalents) activated by HOBt (3 equivalents), DIC (3 equivalents), or HBTU (3 equivalents), DIEA (6 equivalents) in DMF; b) Piperidine/DMF (1∶9) for Fmoc deprotection; ***iv*** TFA-scavenger cocktail (91%), water (3%), triisopropylsilane (3%), and thioanisole (3%) for 4 hr.

Truncated analogs of 2at-LIGRL-*PEG*_3_-*Hdc* **1–6** and compounds **11–14** were prepared as previously published by solid-phase synthesis as summarized in **Figure** **1** on 4-(4-formyl-3-methoxyphenoxy)butyrylaminomethyl resin (aldehyde resin; 0.9 mmol/g) using Fmoc/*t*Bu synthetic strategy and standard DIC-HOBt and HBTU activations [28]. Compounds **7**–**10** were prepared on Rink amide resin (0.67 mmol/g). The synthesis was performed in fritted syringes using a Domino manual synthesizer obtained from Torviq (Niles, MI). All compounds were fully deprotected and cleaved from the resin by treatment with 91% trifluoroacetic acid (TFA; 3% water, 3% triisopropylsilane, and 3% thioanisole). After ether extraction of scavengers, compounds were purified by reverse-phase HPLC and/or size-exclusion chromatography (Sephadex G-25, 0.1 M acetic acid) to >95% purity. Compounds were analyzed for purity by analytical HPLC and MS by Electrospray Ionization or Matrix Assisted Laser Desorption Ionization - Time of Flight (ESI, MALDI-TOF; see below).

### Reductive alkylation (Figure 1, step i)

The aldehyde resin was swollen in dichloromethane (DCM) for 2 hr, washed with DCM and 5% acetic acid in DCM. A mixture of hexadecyl amine (*Hdc*-NH_2_) (5 equivalents), Sodium cyanoborohydride (5 equivalents) in 5% acetic acid in DCM (0.25 M solution) was injected into the syringe reactor. The reaction mixture was stirred overnight. The resin was washed with DCM, 5% acetic acid in DCM, N,N’-dimethylformamide (DMF), 10% diisopropylethylamine (DIEA) in DMF, DCM. A small sample of the secondary amine resin was protected by an Fmoc group (∼30 mg of resin was treated with 10 equivalents of Fmoc-Cl, 10 equiv DIEA in DCM). Resin loading was assessed spectrophometrically (UV at 301 nm; 0.49 mmol/g).

### Solid Phase Synthesis (Figure 1, steps ii-iii)

The aliquot of secondary amide resin from the previous step (10 µmol) was swollen in DCM, washed with tetrahydrofuran-DCM, and the Fmoc-*PEG* was coupled via symmetrical anhydride (6 equiv of N^α^-Fmoc-*PEG* and 3 equivalents of DIC in tetrahydrofuran-DCM) overnight. An on-resin test using Bromophenol Blue was used for qualitative and continuous monitoring of reaction progress. Fmoc group was removed with 10% piperidine in DMF (2 min + 20 min). The resin was washed with DMF (3X), DCM (3X), 0.2 M HOBt in DMF (2X), and finally with DMF (2X). Fmoc-*PEG* and following Fmoc-protected amino acid were coupled using pre-activated 0.3 M HOBt ester in DMF (3 equiv of N^α^-Fmoc amino acid or Fmoc-*PEG*, 3 equivalents of HOBt and 3 equivalents of DIC) monitored by Bromophenol Blue test. To avoid deletion sequences and slower coupling rate in longer sequences, the double coupling was performed at all steps with 3 equivalents of amino acid or Fmoc-*PEG*, 3 equivalents of HBTU and 6 equivalents of DIEA in DMF. Wherever beads still tested Kaiser positive, a third coupling was performed using the symmetric anhydride method (2 equivalent of amino acid and 1 equivalents of DIC in DCM). Any unreacted NH_2_ groups on the resin thereafter were capped using an excess of 50% acetic anhydride in pyridine for 5 min. When the coupling reaction was finished, the resin was washed with DMF, and the same procedure was repeated for the next amino acid until all amino acids were coupled. 2-aminothiazole-4-carboxylic acid and 5-isoxazole-carboxylic acid were attached to the resin as symmetrical anhydride (6 equivalents of acid and 3 equivalents of DIC in DCM-DMF).

### Cleavage of Ligand from the Resin (Figure 1, step iv)

A cleavage cocktail (10 mL per 1 g of resin) of TFA (91%), water (3%), triisopropylsilane (3%), and thioanisole (3%) was injected into the resin and stirred for 4 hr at room temperature. The crude ligand was isolated from the resin by filtration, the filtrate was reduced to low volume by evaporation using a stream of nitrogen, and the ligand was precipitated in ice-cold diethyl ether, washed several times with ether, dried, dissolved in water and lyophilized to give off-white solid powders that were stored at −20°C until purified. The crude compound was purified by size-exclusion chromatography and preparative HPLC.

### Analytical Evaluation

The purity of products was checked by analytical Reverse Phase-HPLC using a Waters Alliance 2695 Separation Model with a Waters 2487 dual wavelength detector (220 and 280 nm) on a reverse phase column (Waters Symmetry C18, 4.6×75 mm, 3.5 µm). Compounds were eluted with a linear gradient of aqueous CH_3_CN/0.1% CF_3_CO_2_H at a flow rate of 1.0 mL/min. Purification of ligands was achieved on a Waters 600 HPLC using a reverse phase column (Vydac C18, 15–20 µm, 22×250 mm). Peptides were eluted with a linear gradient of CH_3_CN/0.1% CF_3_CO_2_H at a flow rate of 5.0 mL/min. Separation was monitored at 230 and 280 nm. Size exclusion chromatography was performed on a borosilicate glass column (2.6×250 mm, Sigma, St. Louis, MO) filled with medium sized Sephadex G-25 or G-10. The compounds were eluted with an isocratic flow of 1.0 M aqueous acetic acid. The pure compounds were dissolved in deionized water or dimethylsulfoxide at approximately 1 mM concentrations. Structures were characterized by ESI (Finnigan, Thermoquest Liquid Chromatography-Quadruplet ion trap instrument) or MALDI-TOF (Bruker Reflex-III) with α-cyanocinnamic acid as a matrix). For internal calibration an appropriate mixture of standard peptides was used with an average resolution of 8,000–9,000. High resolution mass measurements were carried out on a Bruker Ultraflex MALDI TOF-TOF and an Apex Qh Fourier Transformation-Ion Cyclotron Resonance (9.4 T) high resolution instrument.

### Tissue culture

16HBE14o- cells, a SV40 transformed human bronchial epithelial cell line [29], were obtained through the California Pacific Medical Center Research Institute (San Francisco, CA, USA). Cells were maintained and expanded as previously described [19]. Briefly, cell lines were expanded in tissue culture flasks prior to transfer to 96 well E-plates (Roche) for experiments. Flasks and 96 well E-plates were coated initially with a matrix coating solution (88% Lechner and LaVeck basal medium, 10% bovine serum albumin (BSA; from 1 mg/ml stock), 1% bovine collagen type I (from 2.9 mg/ml stock), and 1% human fibronectin (from 1 mg/ml stock solution) and incubated for 2 hr at 37°C, after which the coating solution was removed and allowed to dry for at least 1 hr. 16HBE14o- cells were plated at a concentration of 1×10^5^ cells/cm^2^ and grown in Eagle’s Minimal Essential Medium supplemented with 10% Fetal Bovine Serum (FBS), 2 mM glutamax, penicillin and streptomycin (growth medium) at 37°C in a 5% CO_2_ atmosphere. Growth medium was replaced every other day until the cells reached confluence (5–7 days). Cells were then transferred to 96 well E-plates for RTCA, or collagen/fibronectin/BSA coated glass coverslips for Ca^2+^ imaging experiments.

Primary mouse tracheal epithelial (MTE) cells were cultured as described [28]. Briefly, mouse tracheas were removed, washed in phosphate-buffered saline for 5 min at room temperature, cut lengthwise, and transferred to collection medium [1∶1 mixture of Dulbecco’s Modified Eagle Medium (DMEM) and Ham’s F12 with 1% penicillin-streptomycin) at 37°C. Tracheae were then incubated at 37°C for 2 hr in dissociation medium (44 mM NaHCO_3_, 54 mM KCl, 110 mM NaCl, 0.9 mM NaH_2_PO_4_, 0.25 µM FeN_3_O_9_, 1 µM sodium pyruvate, and 42 µM phenol red, pH 7.5; supplemented with 1% penicillin-streptomycin and 1.4 mg/ml pronase). Enzymatic digestion was stopped by adding 20% FBS. Epithelial cells were gently scraped from the tracheas, centrifuged at 100 x g for 5 min at room temperature. Cell pellets were washed in base culture medium (1∶1 mixture of DMEM and Ham’s F12 with 1% penicillin-streptomycin and 5% FBS) and centrifuged at 100 x g for 5 min at room temperature. MTE cells were resuspended in full culture medium (1∶1 mixture of DMEM and Ham’s F-12, 1% penicillin-streptomycin, 5% FBS, 15 mM Hepes, 3.6 mM sodium bicarbonate, 4 mM L-glutamine, 10 µg/mL insulin, 5 µg/mL transferrin, 25 ng/mL epidermal growth factor, 30 µg/mL bovine pituitary extract) and transferred to collagen/fibronectin/BSA coated tissue culture flasks. Cells were fed every other day for one week until transferred to 96-well E-plates for RTCA experiments.

CHO cells were cultured in DMEM supplemented with 10% FBS and 1% penicillin and streptomycin at 37°C in a 5% CO_2_ atmosphere. One day before transfection, CHO cells were plated in a 60 mm cell culture dish at a concentration of 1×10^5^ cells/cm^2^ and grown without antibiotics. An MrgprC11 cDNA in pcDNA3.1 vector was transfected into CHO cells using Lipofectamine™ 2000 (Invitrogen) prior to transfer to coverslips for experiments. Coverslips were coated with 0.1 mg/ml poly-D-lysine (from 2 mg/ml stock) and incubated for 1 hr at room temperature, after which the coating solution was removed and the coverslips were washed twice with double distilled water. CHO cells were seeded on coverslips at a concentration of 1×10^5^ cells/cm^2^ 6 hr after transfection. Transfected cells were incubated for 24 hr prior to Ca^2+^ imaging.

### *In vitro* physiological response screening

16HBE14o- cells on E-plates in growth medium and in a 37°C, 5% CO_2_ incubator were monitored for the establishment for relative impedance overnight every 15 min using the xCELLigence™ Real Time Cell Analyzer (RTCA, Roche) [19], [28], [30]. When cells reached baseline impedance the next day, and prior to the experiment, the RTCA was moved to room air and temperature where full growth medium was replaced with 100μL modified Hank’s Balanced Saline Solution (HBSS) pre-warmed to 37°C. The RTCA was then allowed to come to room temperature (45–60 min) prior to ligand addition. Each well was then supplemented with 100 µL HBSS containing appropriate ligands to measure concentration response ranges in quadruplicate. Additional wells were used for vehicle controls. Relative impedance in each well was monitored every 30 sec over 4 hr. Peak responses, defined as the maximal change in Normalized Cell Index, were used to define maximal response concentrations and physiological EC_50_s for each ligand.

The use of primary cultured MTE cells required different treatments and resulted in reduced overall signaling. Briefly, MTE cells were transferred to E-plates in minimal culture medium (100 µL/well of 1∶1 mixture of DMEM and Ham’s F-12, 1% penicillin-streptomycin, 3.6 mM sodium bicarbonate, 4 mM L-glutamine) and allowed to adhere for 4 hr. At that time each well was supplemented with 2x concentration of agonist in minimal culture medium. Relative impedance in each well was monitored every 30 sec for up to 2 hr.

### *In vitro* Ca^2+^ Imaging

16HBE14o- cells or CHO cells were loaded with fura 2-acetomethoxyl ester (CalBiochem or Molecular Probes) for 30 min at room temperature. Cells were washed with HBSS and allowed to sit for at least 20 min prior to digital imaging. For activation and desensitization assay experiments using 16HBE14o- cells, [Ca^2+^]_i_ was measured as previously described [19]. Experiments consisted of 20 sec of recording of cells in HBSS to determine resting [Ca^2+^]_i,_ followed by a 10 sec wash to introduce ligand and up to 10 min of recording for ligand washes required for desensitization experiments. Briefly, fura-2 fluorescence was observed on an Olympus IX70 microscope with a 40X oil immersion objective after alternating excitation between 340 and 380 nm by a 75 W Xenon lamp linked to a Delta Ram V illuminator (PTI) and a gel optic line. Intracellular Ca^2+^ concentration ([Ca^2+^]_i_) for each individual cell in the field of view was calculated by ratiometric analysis of fura-2 fluorescence using equations originally published in [31]. Individual ratios were calculated every sec throughout the experiments. [Ca^2+^]_i_ traces over time are averaged [Ca^2+^]_i_ of all cells within a field of view (80–120) and are representative of at least 3 experiments. Cells were considered activated by the ligand if their resting [Ca^2+^]_i_ was increased above a 200 nM threshold, a 2–4 fold increase above typical resting values. Percent activation graphs are determined from 3–6 experiments for each ligand concentration. Time to threshold in individual cells represent between 200–400 cells from at least 3 experiments at each ligand concentration. Ca^2+^ imaging of CHO cells was similar; each ligand concentration tested included at least 3 experiments and a minimum of 200 cells analyzed during each experiment.

### *In vivo* mechanical sensitivity

Male ICR mice (Harlan) or PAR_2_^-/-^ mice and their wild type littermates on a C57Bl/6 background weighing 25–30 grams were used for these studies. Animal protocols were approved by the Institutional Animal Care and Use Committee of The University of Arizona. Compounds were injected into the plantar surface of the hindpaw in a total volume of 25 µL using a 31-gauge needle. Compounds were diluted using sterile saline. Mechanical thresholds were determined using calibrated von Frey filaments (Stoelting Co, Wood Dale, IN) with the up-down method [32]. The experimenter was always blinded to the treatment conditions and animals were randomized such that animals in a single experimental group were never all housed together.

### Statistical Analysis

All statistical analyses were evaluated with GraphPad software (San Diego, CA). Multivariate comparisons were done with a two-way ANOVA with Tukey's or Bonferroni multiple comparison post-test as appropriate for the individual experiment. Pair-wise comparisons were done with a two-tailed Student's *t*-test. A value of p<0.05 was used to establish a significant difference between samples. Data in Figures are graphed ± Standard Error of the Mean (SEM) unless otherwise noted.

## Results

### Determination of a minimal peptidomimetic structure needed for PAR_2_ activation using synthetic tethered ligands (STLs)

#### STL construction

Truncated analogs of 2at-LIGRL-*PEG*_3_-*Hdc*, compounds **1**–**6,** were synthesized using standard Fmoc chemistry on aldehyde amino methyl resin as described in **Figure 1** and in detail in Flynn, et al [28]. Briefly, compounds assembled on the solid support were cleaved from the resin with TFA-scavenger cocktails and purified by Reverse Phase-HPLC and/or size-exclusion chromatography. All compounds gave > 95% analytical HPLC and expected MS (data not shown).

#### In vitro potency

**Figure 2:**
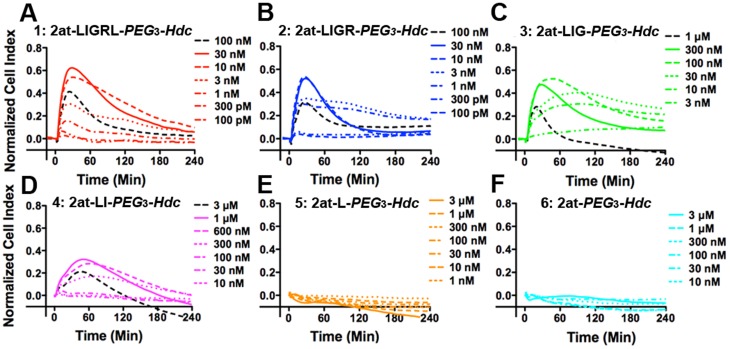
*In vitro* physiological responses of 16HBE14o- cells following addition of STL agonist compounds 1–6. Each panel (**A–F**) represents physiological response, measured using xCELLigence™ RTCA and expressed as a Normalized Cell Index over time following addition of STL. (**A**) Compound **1**, 2at-LIGRL-*PEG*_3_-*Hdc*; (**B**) Compound **2**, 2at-LIGR-*PEG*_3_-*Hdc*; (**C**) Compound **3**, 2at-LIG-*PEG*_3_-*Hdc*; (**D**) Compound **4**, 2at-LI-*PEG*_3_-*Hdc*; (**E**) Compound **5**, 2at-L-*PEG*_3_-*Hdc*; (**F**) Compound **6**, 2at-*PEG*_3_-*Hdc*. Concentrations for each experiment chosen to highlight supramaximal (dashed black traces), maximal (solid traces) and concentration dependent responses are shown at right of individual plots. Traces are averages from three or four experiments and are representative of experiments from at least two independent E-plates. Standard deviations have been removed to promote clarity. Systematic truncation of the parent peptidomimetic reduces agonist response starting with compound **3** until no activity remains in compounds **5** and **6**. The reduced peak responses at supramaximal and maximal concentrations of compound **4** are suggestive of a partial agonist.

**Figure 3:**
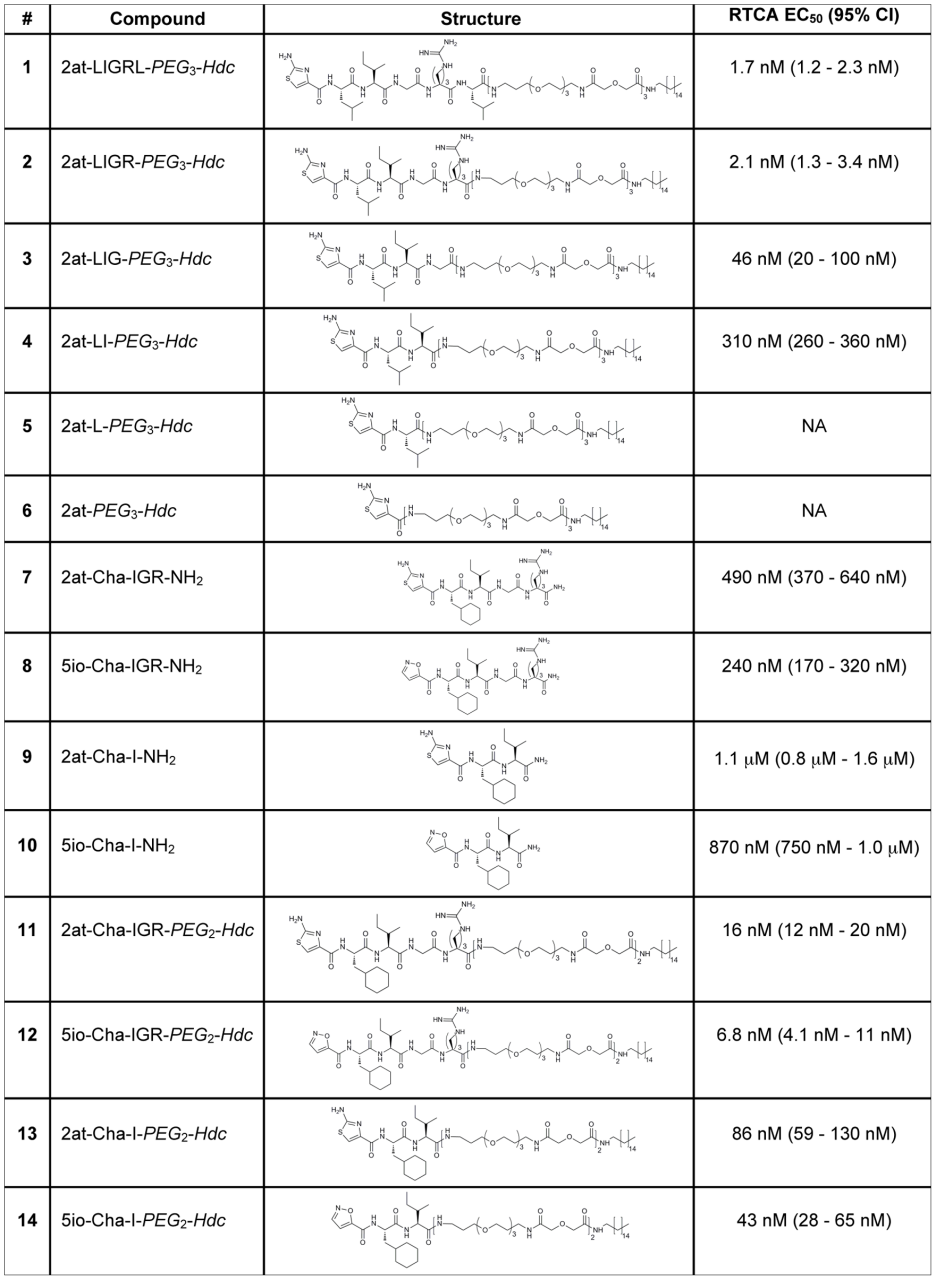
PAR_2_ ligand structures and RTCA EC_50_. Compound number, name, structure and the *in vitro* physiological EC_50_ (RTCA) of each compound described in the manuscript are shown for comparison. #, compound number; Name, compound name; Structure, compound structure; RTCA, xCELLigence™ real time cell analysis; EC_50_, half maximal effective concentration; 95% CI, 95% confidence interval.

**Figure 4:**
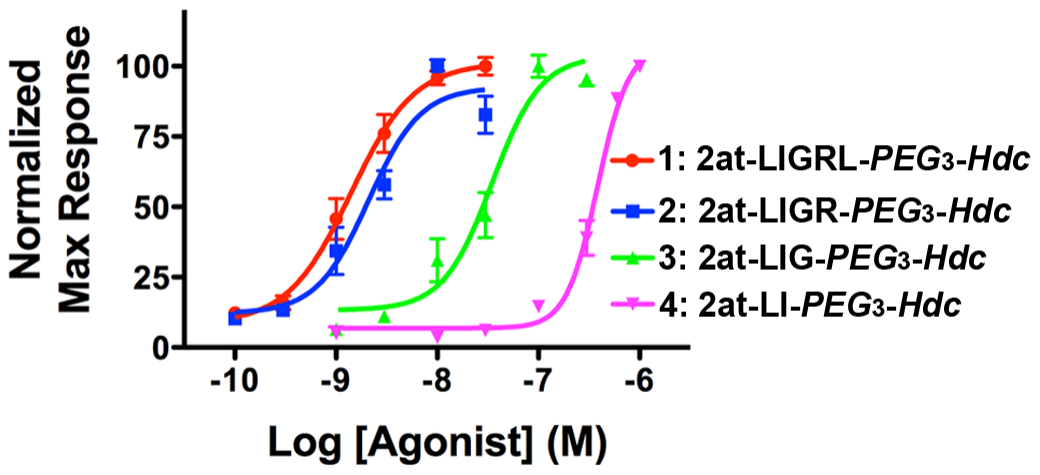
Concentration response curves for STL agonist compounds 1–4. Concentration response curves were developed from i*n vitro* physiological responses (RTCA) using the peak response within the 4 hr experiment. Compounds **1** (2at-LIGRL-*PEG*_3_-*Hdc*) and **2** (2at-LIGR-*PEG*_3_-*Hdc*) have roughly equivalent EC_50_s (see **Figure 3**), while further truncated compounds **3** (2at-LIG-*PEG*_3_-*Hdc*) and **4** (2at-LI-*PEG*_3_-*Hdc*) have higher EC_50_s (see **Figure 3**).

Compounds **1–6** were first evaluated for *in vitro* physiological response using the xCELLigence™ RTCA. RTCA measures physiological interactions between the cellular membrane and a surface substrate using underlying electrodes that register changes in impedance (reported as a Cell Index) over a prolonged time course in a non-invasive system and has been used to evaluate PAR_2_ agonist potency [19], [28], [30]. Each compound was applied to the cells over an appropriate concentration range and Cell Index was monitored over a 4 hr experiment. Agonist activity was similar between compound **1**, the full length STL (2at-LIGRL-*PEG*_3_-*Hdc*; previously published as Compound **12** in Flynn, et al. [28]), and an STL missing the C-terminal Leu_6_, compound **2** (2at-LIGR-*PEG*_3_-*Hdc*), in their respective physiological responses (**Figure 2**) and in their calculated EC_50_s (compound **1** EC_50_ = 1.7 nM, 95% CI: 1.2–2.3 nM; **2** EC_50_  = 2.10 nM, 95% CI: 1.3–3.4 nM; **Figure 3**). Similar to previous RTCA analyses of PAR_2_ agonists, maximal response concentrations (solid lines in **Figure 2**) displayed faster return to baseline, and supramaximal concentrations (dashed black lines) resulted in reduced peak responses and faster returns to baseline [19], [28]. Compound **3** (2at-LIG-*PEG*_3_-*Hdc*), constructed without two amino acids from the C-terminus of compound **1**, displayed a reduced response in the RTCA assay (**Figure 2**) and a reduced EC_50_ (46 nM, 95% CI: 20–100 nM; **Figure 3**). Compound **4** (2at-LI-*PEG*_3_-*Hdc*), missing 3 amino acids from C-terminus of compound **1**, was further reduced in RTCA response (**Figure 2**) and potency (EC_50_ = 310 nM: 95% CI 260–360 nM; **Figure 3**). The maximal response concentration of compound **4** also failed to reach a peak response similar to known full agonists 2-furoyl-LIGRLO-NH_2_, 2at-LIGRL-NH_2_ [19], [28] or compounds **1–3,** consistent with partial agonism of PAR_2_. Further C-terminus truncation (compounds **5** (2at-L-*PEG*_3_-*Hdc*) and **6** (2at-*PEG*_3_-*Hdc*)) eliminated ligand activity as measured by RTCA (**Figure 2**). Comparison of concentration response curves for compounds **1–4** shows relative equal potency for compounds **1** and **2**, and measurable loss of potency (right shifts) for compounds **3** and **4** (**Figure 4**).

#### Specificity of PAR_2_ agonists

**Figure 5:**
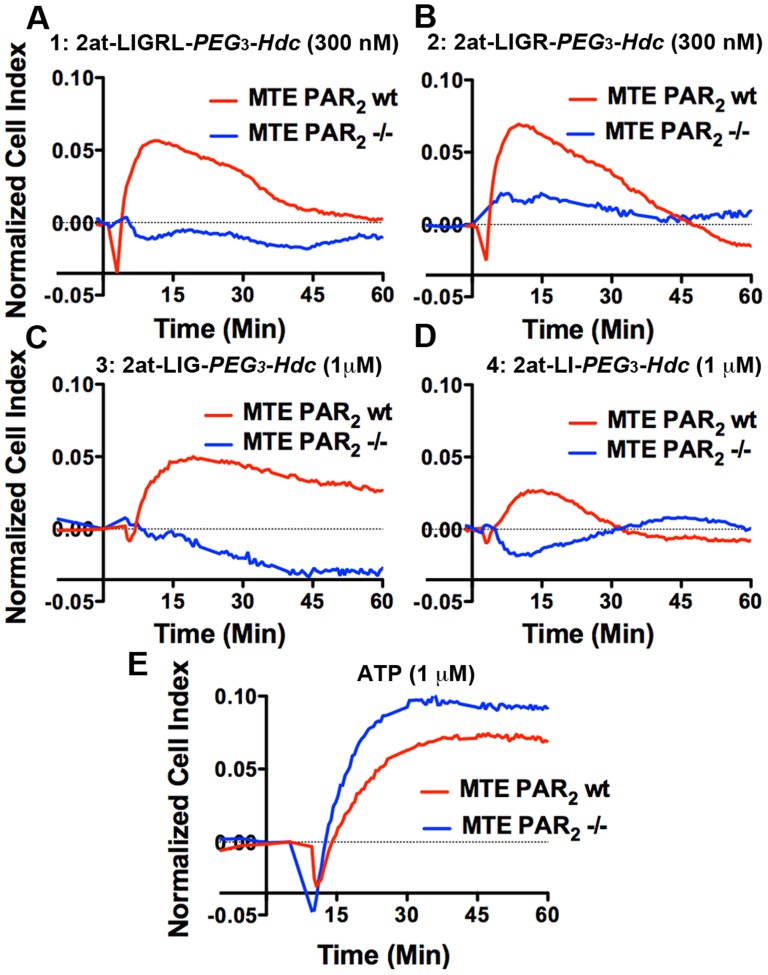
*In vitro* physiological responses of MTE cells following addition of STL agonist compounds 1–4. Each panel (**A–E**) compares physiological response, measured with xCELLigence™ RTCA as a Normalized Cell Index over time following addition of STL in MTE cells cultured from PAR_2_ expressing mice (PAR_2_ wt, red traces) or PAR_2_ null mice (PAR_2_^-/-^, blue traces). Concentrations for each experiment are shown with each STL agonist (compounds **1–4)** and a PAR_2_-independent agonist, ATP. Traces are averages from three or four experiments and are representative of experiments from at least two independent E-plates. Standard deviations have been removed to promote clarity. PAR_2_ expression is required for response to compounds **1–4**, but not for response to ATP.

Although the RTCA experiments using 16HBE14o- cells provide a highly sensitive physiological assay that encompasses various cell signaling responses to an agonist, it is inherently limited in detecting receptor specificity. To further evaluate specificity of known and novel STLs, we first compared RTCA responses to compounds **1–4** using primary cultured mouse tracheal epithelial (MTE) cells from wild type and PAR_2_^-/-^ mice ([28]; **Figure 5**). The effective Cell Index for peak concentration response for each compound **1–4** was reduced in MTE cultures when compared to the 16HBE14o- cells. More importantly, compounds **1–4** all required PAR_2_ expression to establish RTCA responses. Also similar to the 16HBE14o- RTCA traces, compound **4** displayed a reduced peak response in the PAR_2_-expressing MTE. To demonstrate signaling competence in both cultures, stimulation with the PAR_2_ independent agonist ATP resulted in similar RTCA responses in both wild type and PAR_2_^-/-^ primary MTE cultures.

**Figure 6:**
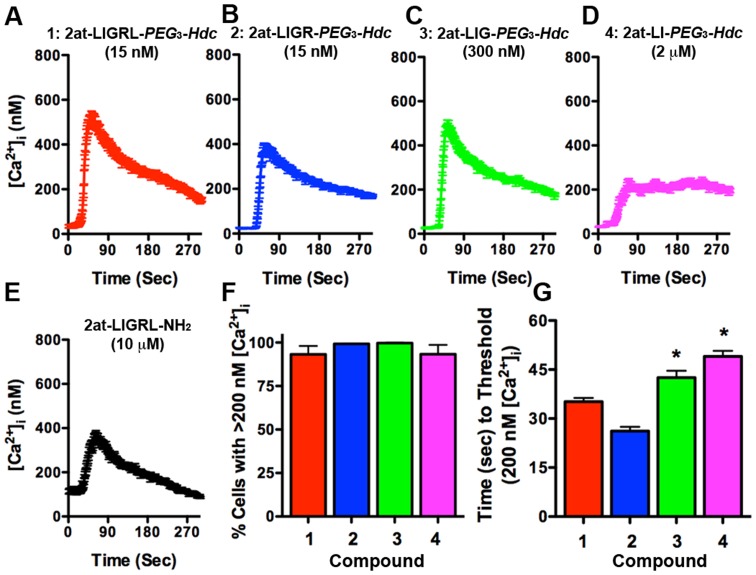
Ca^2+^ signaling responses for STL agonist compounds 1–4. The top four panels (**A–D**) display traces from a single experiment of average individual cell [Ca^2+^]_i_ (± SEM) over time for 16HBE14o-cells exposed to PAR_2_ STL agonist compounds **1–4**. Concentrations (Compound **1**: 2at-LIGRL-*PEG*_3_-*Hdc*, 15 nM; **2**: 2at-LIGR-*PEG*_3_-*Hdc*, 15 nM; **3**: 2at-LIG-*PEG*_3_-*Hdc*, 200 nM; **4**: 2at-LI-*PEG*_3_-*Hdc*, 2 µM) were chosen to reflect minimal agonist concentration necessary to result in 95% activation of 16HBE14o- cells (n≥3 for each compound). The known peptidomimetic full agonist, (**E**) 2at-LIGRL-NH_2_ (10 µM) is shown for comparison. Although all compounds displayed full Ca^2+^ activation over the 5 min experiment (**F**), compounds **3** and **4** displayed a slight delay in average time to peak Ca^2+^ response (**G**).

Because [Ca^2+^]_i_ changes are a primary outcome following PAR_2_ activation, we further tested for PAR_2_ specificity using a Ca^2+^ desensitization assay with the known PAR_2_ agonist, 2at-LIGRL-NH_2_ [19], [33]. Using digital imaging microscopy, we first evaluated minimal ligand concentrations that would induce 90–100% activation in 16HBE14o- cells within a 5 min experiment (**Figure 6**). Sample traces of average [Ca^2+^]_i_ changes plotted over time for compounds **1–4** and the parent peptidomimetic, 2at-LIGRL-NH_2_ are consistent with RTCA recordings. Compounds **1** and **2** were highly potent ligands (15 nM) whereas compound **3** (300 nM) required higher concentrations to elicit the full Ca^2+^ response, albeit with a significant delay in the time required to reach threshold [Ca^2+^]_i_ when compared with compounds **1** and **2 (** **Figure 6F, G** **)**. Compound **4** required even higher concentrations (2 µM) to achieve threshold [Ca^2+^]_i_ changes. Even at this heightened concentration compound **4** displayed a significant drop in average peak [Ca^2+^]_i_ as well as lack of return to baseline [Ca^2+^]_i_ within the 5 min experiment (**Figure 6D**).

**Figure 7:**
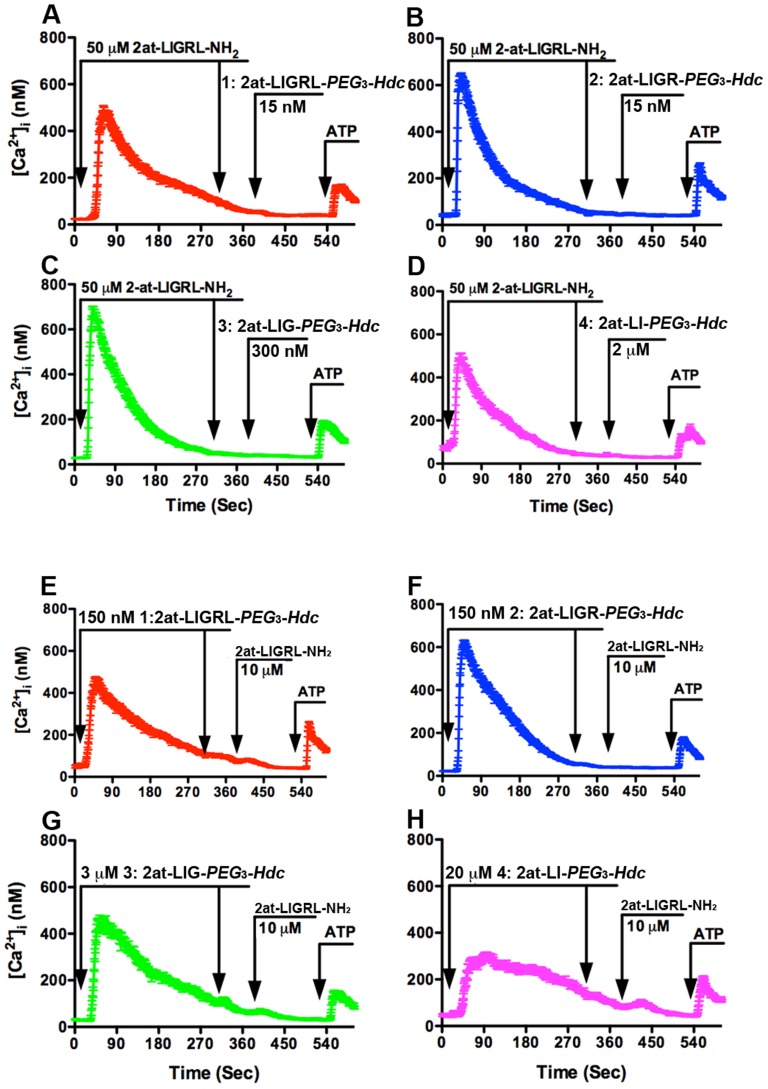
Ca^2+^ desensitization responses for STL agonist compounds 1–4. The top four panels (**A–D**) display traces of the average change in [Ca^2+^]_i._ for all cells in the field of view plotted over time (10 min). In each panel, PAR_2_ desensitization with 50 µM 2at-LIGRL-NH_2_ prevented Ca^2+^ signaling by a second application of 2at-LIGRL-NH_2_ and subsequent addition of PAR_2_ STL agonists — Compound **1**: 2at-LIGRL-*PEG*_3_-*Hdc*, 15 nM; **2**: 2at-LIGR-*PEG*_3_-*Hdc*, 15 nM; **3**: 2at-LIG-*PEG*_3_-*Hdc*, 200 nM; **4**: 2at-LI-*PEG*_3_-*Hdc*, 2 µM. Subsequent application of 5 µM ATP in each experiment demonstrated that Ca^2+^ response was intact, and only PAR_2_ dependent pathways were desensitized. In the bottom four panels (**E–H**), 16HBE14o- cells were desensitized with the STL compounds **1–4** at 10 fold their full activation concentrations Compound **1**: 2at-LIGRL-*PEG*_3_-*Hdc*, 150 nM; **2**: 2at-LIGR-*PEG*_3_-*Hdc*, 150 nM; **3**: 2at-LIG-*PEG*_3_-*Hdc*, 2 µM; **4**: 2at-LI-*PEG*_3_-*Hdc*, 20 µM). Although compounds **1–3** were fully effective in desensitizing the cells to 10 µM 2at-LIGRL-NH_2_, desensitization by compound **4** was not complete. In each case, responses to 5 µM ATP remained fully in tact.

For desensitization studies, 16HBE14o- cells were first exposed to a high concentration of 2at-LIGRL-NH_2_ to effectively eliminate PAR_2_ based signaling prior to application of compounds **1–4**. Thus, any increase in [Ca^2+^]_i_ in response to compounds **1–4** would indicate a response that was not specific to PAR_2_. When 16HBE14o- cells were desensitized with 50 µM 2at-LIGRL-NH_2_, a second wash with 50 µM 2at-LIGRL-NH_2_ did not result in an increase of [Ca^2+^]_i_ (**Figure 7A–D**). Subsequent treatment with any of the four compounds also did not result in measurable changes in [Ca^2+^]_i_. This loss of response was not caused by loss of Ca^2+^ signaling itself, as ATP remained an effective agonist following desensitization of PAR_2_. We further tested the agonist ability of these novel PAR_2_ agonists by using 10 fold the fully activating concentration for each compound to desensitize 16HBE14o- responses to 10 µM 2at-LIGRL-NH_2_ (**Figure 7E–H**). In these experiments, compounds **1–3** effectively desensitized 16HBE14o- cells, however, compound **4** could not fully eliminate the 2at-LIGRL-NH_2_-induced Ca^2+^ response.

**Figure 8:**
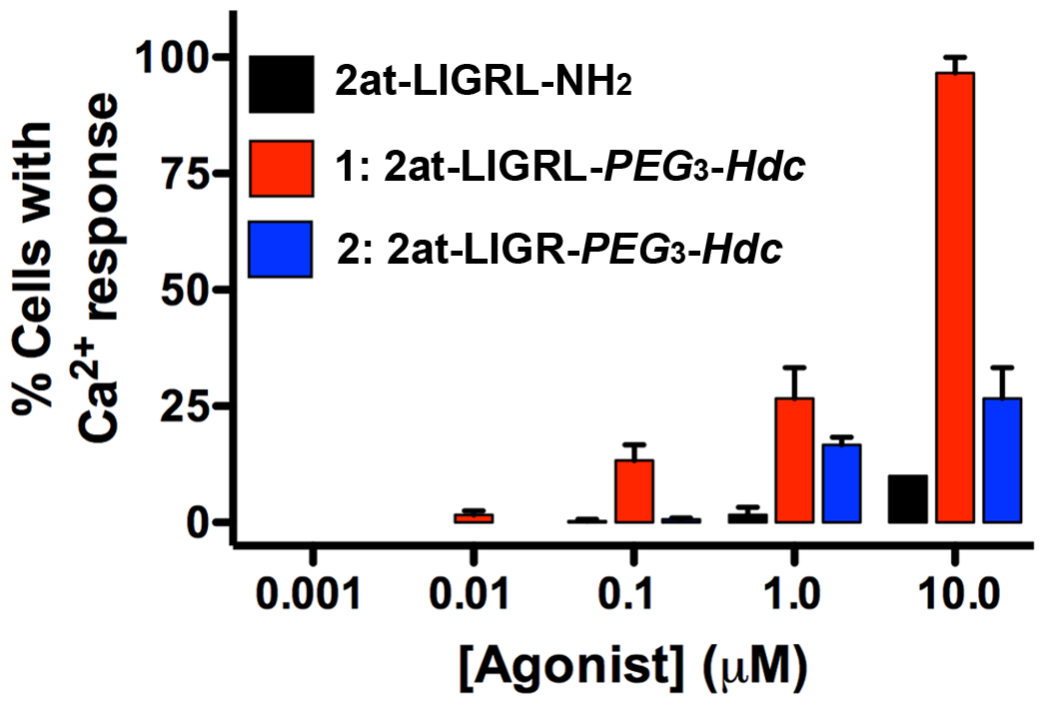
Selectivity of parent compound 2at-LIGRL-NH_2_ and potent STL agonist compounds 1, 2 for PAR_2_ over MrgprC11. Compounds were applied to PAR_2_ deficient CHO cells transfected with MrgprC11 and evaluated for Ca^2+^ response. Compound **1**: 2at-LIGRL-*PEG*_3_-*Hdc* was able to induce a full Ca^2+^ response at the highest concentration tested that was negligible concentrations typically used for PAR_2_ activation (e.g., 1**–**10 nM). Both the parent compound (2at-LIGRL-NH_2_) and the Leu_6_-truncated STL (compound **2**: 2at-LIGR-*PEG*_3_-*Hdc*) displayed limited MrgprC11 activity. None of the compounds displayed activity in untransfected CHO cells (not shown). Each column represents three experiments, each with ∼200 cells.

It has been demonstrated that MrgprC11 can be activated by the PAR_2_ peptide agonist SLIGRL-NH_2_, (EC_50_ = 10 µM) however, the Leu_6_-truncated peptide, SLIGR-NH_2_, lost MrgprC11 signaling capacity while retaining PAR_2_ activity [12]. To examine PAR_2_/MrgprC11 selectivity, we evaluated Ca^2+^ responses in MrgprC11 transfected CHO cells [12] with the parent peptidomimetic, 2at-LIGR-NH_2_ and the most potent STL compounds (**1**, **2**) from this study (**Figure 8**). When 2at-LIGRL-NH_2_ was applied to MrgprC11 transfected CHO cells at very high concentration (10 µM), a modest Ca^2+^ response was observed (10% of cells in the field of view); no response was observed at 1 µM. In contrast, 10 µM of the full length PAR_2_-STL, compound **1**, induced a robust Ca^2+^ response (88 ± 19%) in the MrgprC11 transfected cells. This Ca^2+^ response decreased profoundly at concentrations of compound **1** tested at 100 times higher than the RTCA EC_50_ of ∼ 1 nM (20% response at 1 µM, 10% response at 100 nM, 2.5% response at 10 nM, 0% at 1 nM). In contrast, the potent PAR_2_ agonist compound **2** (2at-LIGR-*PEG*_3_-*Hdc*) induced limited Ca^2+^ responses in the MrgprC11 transfected cells even at the highest concentrations tested (20% at 10 µM, 15% at 1 µM, 0.5% at 100 nM). All three compounds tested displayed selectivity for PAR_2_ versus MrgprC11 with 2at-LIGRL-NH_2_ and compound **2**, 2at-LIGR-*PEG*_3_-*Hdc*, displaying minimal MrgprC11 activity at concentrations up to 10 µM.

#### *In vivo* efficacy

**Figure 9:**
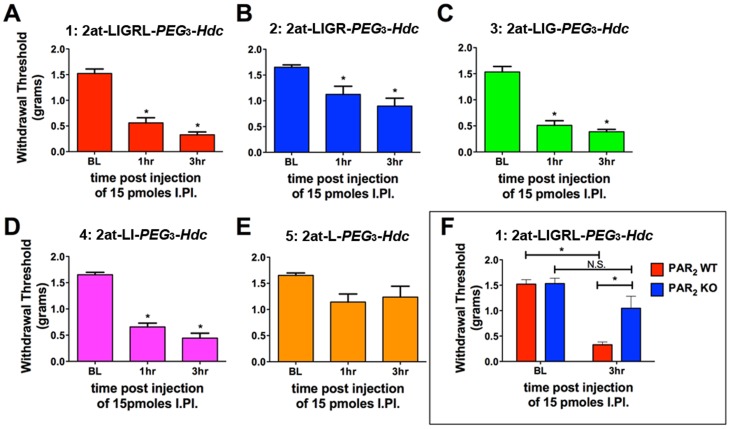
*In vivo* assessment of STL agonist compounds 1–5 induced mechanical hypersensitivity. Compounds were injected into the plantar surface of the hindpaw at 15 pmoles and mechanical sensitivity was measured at 1 and 3 hr following injection. Compounds **1**: 2at-LIGRL-*PEG*_3_-*Hdc*, **2**: 2at-LIGR-*PEG*_3_-*Hdc*, **3**: 2at-LIG-*PEG*_3_-*Hdc* and **4**: 2at-LI-*PEG*_3_-*Hdc* evoked mechanical hypersensitivity whereas Compound **5** (E; 2at-L-*PEG*_3_-*Hdc*) was inactive. Compound **1** caused mechanical hypersensitivity in PAR_2_ WT mice but was inactive in PAR_2_^-/-^ mice (F). * p<0.05.

Stimulation of PAR_2_ *in vivo* is known to promote mechanical sensitization reflected by a mechanical hypersensitivity response in the von Frey test [8], [10], [11]. Compounds **1–5** were individually injected into the hindpaw of mice following evaluation of baseline mechanical sensitivity and mechanical thresholds were evaluated at 1 and 3 hr post-injection. Based on the EC_50_s of parent compounds [19], [28], [32] we utilized a dose of 15 pmoles for experimentation. Consistent with *in vitro* findings, compounds **1–4** evoked mechanical hypersensitivity at 1 and 3 hr following injection (**Figure 9A–D**) whereas compound **5** lacked activity (**Figure 9E**). We did not note any itch response following injection of any tested compound. To determine specificity of these ligands, we tested the parent compound for mechanical hypersensitivity in wild type (WT, C57Bl/6 background) and PAR_2_^-/-^ mice (C57Bl/6 background). Compound **1** (15 pmoles) stimulated mechanical hypersensitivity in WT mice but failed to do so in PAR_2_^-/-^ mice (**Figure 9F**). Therefore, these compounds are specific agonists at PAR_2_ *in vivo* with the minimal peptide sequence *in vivo* matching the *in vitro* activity.

#### Evaluation of novel peptidomimetics using STL and RTCA

**Figure 10:**
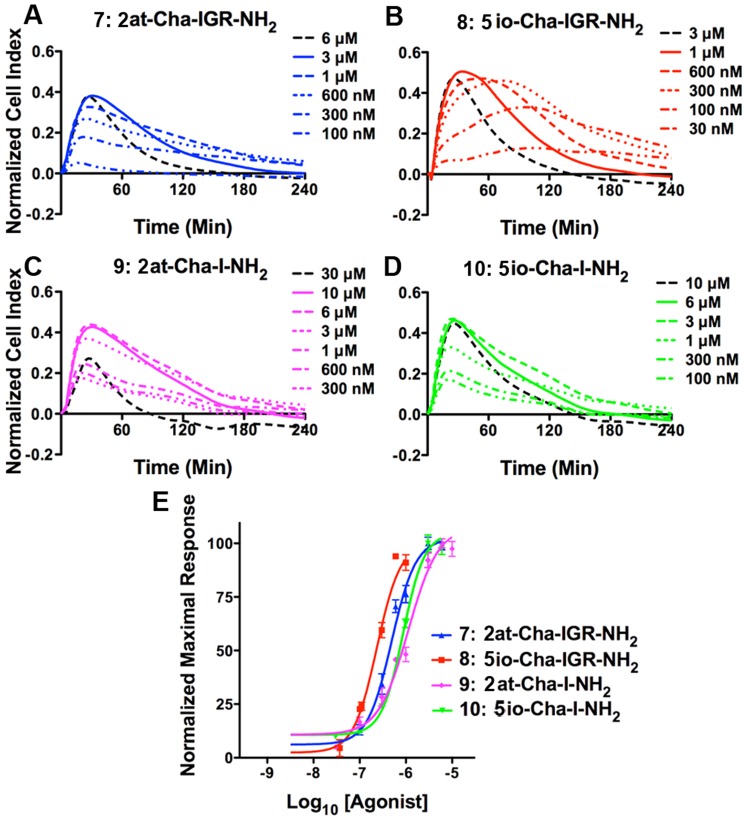
*In vitro* physiological responses of 16HBE14o- cells following addition of agonist compounds 7–10. Each of the top four panels (**A–D**) represents physiological response to agonist compounds as described for **Figure 2**. Concentrations for each experiment (at right of plots) show concentration responses that include supramaximal (black dashed lines) and maximal (solid line) responses. Compound **7**: 2at-Cha-IGR-NH_2_, compound **8**: 5io-Cha-IGR-NH_2_, compound **9**: 2at-Cha-I-NH_2_, and compound **10**: 5io-Cha-I-NH_2_ all display rapid RTCA responses. However, compounds **7**, **9**, and **10**, all exhibit reduced peak Normalized Cell Index responses. Concentration response curves developed from RTCA using the peak response within the 4 hr experiment are shown in the bottom panel. Compounds **7–10** display activity consistent with previously described heterocycle-pentapeptides PAR_2_ agonists [19], [28]. EC_50_s for each compound are shown in **Figure 3**.

A previous report suggested that the heterocycle Ser_1_ substitute isoxazole (io) combined with the amino acids cyclohexylalanine (Cha) and Ile_3_ (e.g., 5io-Cha-I-NH_2_) was sufficient to specifically activate PAR_2_ [21]. However, in that report the authors also noted that full responses of this compound were not available due its lack of solubility and the sensitivity of the chosen Ca^2+^ assay used to evaluate PAR_2_ activation. Based on the previous report and our above data showing equipotency between compounds **1** and **2**, we first looked at tetrapeptide mimetics that included the aminothiazoyl or isoxazole heterocycle paired with a Cha-IGR amino acid sequence and created compounds **7**: 2at-Cha-IGR-NH_2_ and **8**: 5io-Cha-IGR-NH_2_. We followed these with the proposed minimal heterocycle-dipeptides: compounds **9**: 2at-Cha-I-NH_2_ and **10**: 5io-Cha-I-NH_2_ (**Figure 3**). To evaluate potency of these compounds, we first took advantage of the highly sensitive nature of the RTCA *in vitro* physiological response (**Figure 10**). Compounds **7** (EC_50_ = 490 nM, CI: 370–640 nM) and **8** **(**EC_50_ = 240 nM, 95% CI: 170–320 nM) displayed RTCA EC_50_ responses consistent with high activity heterocycle-tetrapeptides, with compound **8** showing the most potent responses recorded for tetra-, penta-, or hexa-peptide mimetics used in this assay (e.g., 2at-LIGRL-NH_2_ and 2-furoyl-LIGRLO-NH_2_; [19]). Compounds **9** and **10** displayed reduced potency RTCA responses (compound **9**: 1.1 µM**,** 95% CI: 810 nM–1.6 µM; **10** EC_5**0**_ = 870 nM, 95% CI: 750 nM–1.0 µM; **Figure 3**). The isoxazole heterocycle demonstrated slightly higher potency when compared with similar length peptidomimetics containing the aminothiazoyl Ser_1_ substitute. Interestingly, from this shortened peptide group with the Leu_2_ Cha substitution, only compound **8** consistently displayed a Normalized Cell Index response consistent with full PAR_2_ agonism in the RTCA assay (**Figure 10**).

**Figure 11:**
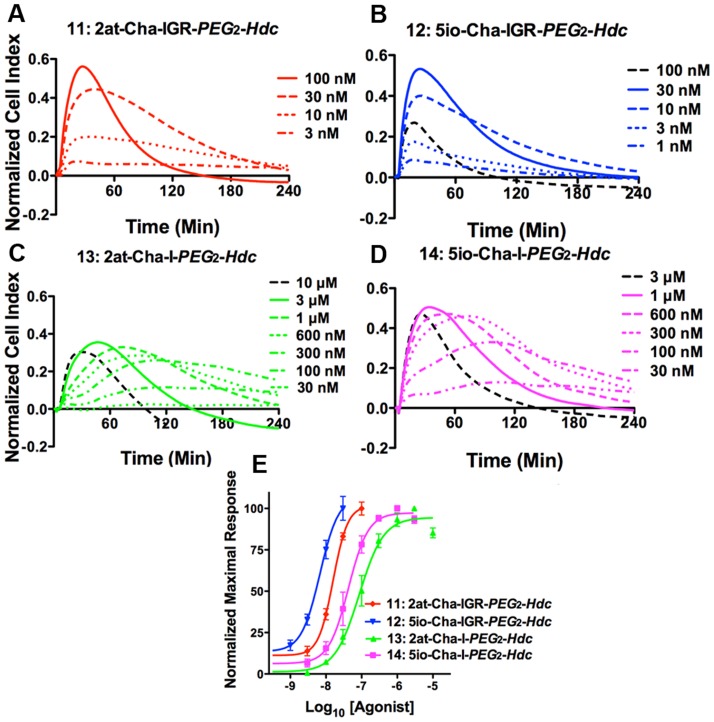
*In vitro* physiological responses of 16HBE14o- cells following addition of STL agonist compounds 11–14. Each of the top four panels (**A–D**) represents physiological responses to agonist compounds as described for **Figure 2**. Concentrations for each experiment (at right of plots) show concentration responses that include supramaximal (black dashed lines) and maximal (solid line) responses. Compounds **11**: 2at-Cha-IGR-*PEG*_2_-*Hdc*, and **12**: 5io-Cha-IGR-*PEG*_2_-*Hdc* both display rapid and full RTCA responses. However, compounds **13**: 2at-Cha-I-*PEG*_2_-*Hdc* and **14**: 5io-Cha-I-*PEG*_2_-*Hdc* display delayed responses across concentration ranges that fall short of peak Normalized Cell Index typical for a full agonist. (**E**) Concentration response curves developed from RTCA using the peak response within the 4 hr experiment are shown in the bottom panel. EC_50_s for each compound are shown in **Figure 3**.

To better characterize differences among these heterocycle-tetrapeptides and heterocycle-dipeptides, we constructed companion STLs with two polyethylene glycol groups and a hexadecyl group (i.e., *PEG*_2_-*Hdc* attached to the C-terminus) and tested them for *in vitro* physiological responses with RTCA (**Figure 11** **,** **Figure 3**). Compounds **11** (2at-Cha-IGR-*PEG*_2_-*Hdc*) and **12** (5io-Cha-IGR-*PEG*_2_-*Hdc*) displayed RTCA EC_50_s in the nM range (EC_50_ = 16 nM, 95% CI: 12–20 nM and EC_50_ = 6.8 nM, 95% CI: 4.1–11 nM, respectively). Similar to the pattern observed above, truncation to the heterocycle-dipeptide STL, compounds **13** (2at-Cha-I-*PEG*_2_-*Hdc*) and **14** (5io-Cha-I-*PEG*_2_-*Hdc*), resulted in less potent agonists (**13**: EC_50_ = 86 nM, 95% CI: 59–130 nM and **14**: EC_50_ = 43 nM, 95% CI: 28–65 nM). Additionally, both compounds **13** and **14** displayed a delayed onset of response that was most prominent at submaximal concentrations. Further, compound **13** clearly did not attain peak Normalized Cell Index responses observed by other compounds in this group. A beneficial outcome of increased sensitivity using the STL construction and RTCA analysis was the separation of potency when comparing compounds that only differed in their respective heterocycle head group. Notably, when Cha was substituted for Leu_2_ the isoxazole containing compounds displayed an increased potency over the aminothiazoyl containing compounds in their peptidomimetic form (compounds **7–10**; **Figure 10E** **,** **Figure 3**) that was only clearly separable when tested in their STL form (compounds **11–14;** **Figure 11E** **,** **Figure 3**).

**Figure 12:**
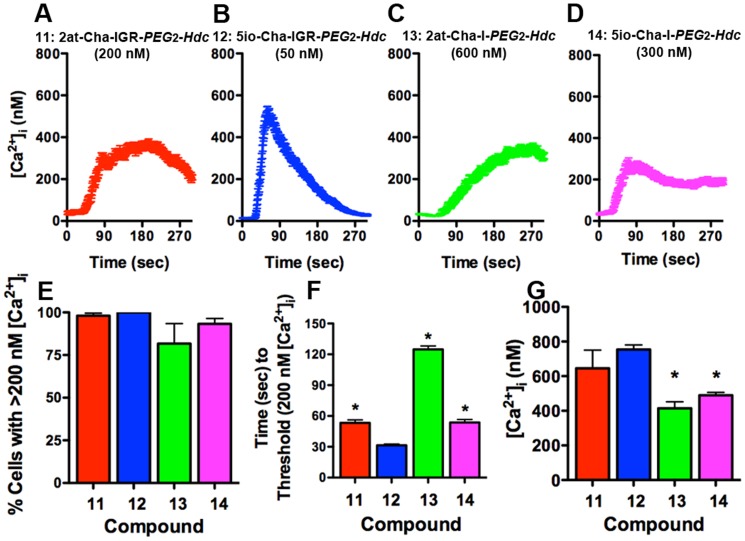
Ca^2+^ signaling responses for STL agonist compounds 11–14. The top four panels (**A–D**) display traces from a single experiment of average individual cell [Ca^2+^]_i_ (± SEM) over time for 16HBE14o-cells exposed to PAR_2_ STL agonist compounds **11–14**. **(E)** Concentrations (Compound **11**: 2at-Cha-IGR-*PEG*_2_-*Hdc*, 200 nM; **12**: 5io-Cha-IGR-*PEG*_2_-*Hdc*, 50 nM; **13**: 2at-Cha-I-*PEG*_2_-*Hdc*, 600 nM; **14**: 5io-Cha-I-*PEG*_2_-*Hdc*, 300 nM) were chosen to reflect minimal agonist concentration necessary to result in the maximal activation of 16HBE14o- cells (n≥3 for each compound). (**F**) Compounds **11**, **13** and **14** all demonstrated significantly delayed responses to Ca^2+^ peak and (**G**) compounds **13** and **14** also displayed significantly reduced peak [Ca^2+^]_i_ changes. Of this group, only compound **12** displayed Ca^2+^ signaling responses representative of full PAR_2_ agonism.

We further characterized compounds **11–14**, using the Ca^2+^ signaling assays. Typical Ca^2+^ traces with average [Ca^2+^]_i_ changes (85–110 cells) plotted over time for compounds **11–14** are shown (**Figure 12A–D**). Concentrations for each compound were established by their ability to elicit 80–100% activation of 16HBE14o- cells above threshold (**Figure 12E**), and were consistent with the RTCA data in that the heterocycle-tetrapeptide STL constructions required lower concentrations than the heterocycle-dipeptide STLs. However, only compound **12** (5io-Cha-IGR-*PEG*_2_ *-Hdc*) elicited Ca^2+^ traces consistent with a full PAR_2_ agonist. Both of the heterocycle-Cha-I-*PEG*_2_ *-Hdc* compounds (**13** and **14**) could not consistently activate >95% of the cells in the 5 min experiment (**Figure 12E**). Examination of Ca^2+^ signaling data showed that compounds **11**, **13** and **14** all exhibited a significantly delayed time to Ca^2+^ threshold following ligand application, and the heterocycle-dipeptide compounds also exhibited a reduced peak [Ca^2+^]_i_ change (**Figure 12F–G**).

**Figure 13:**
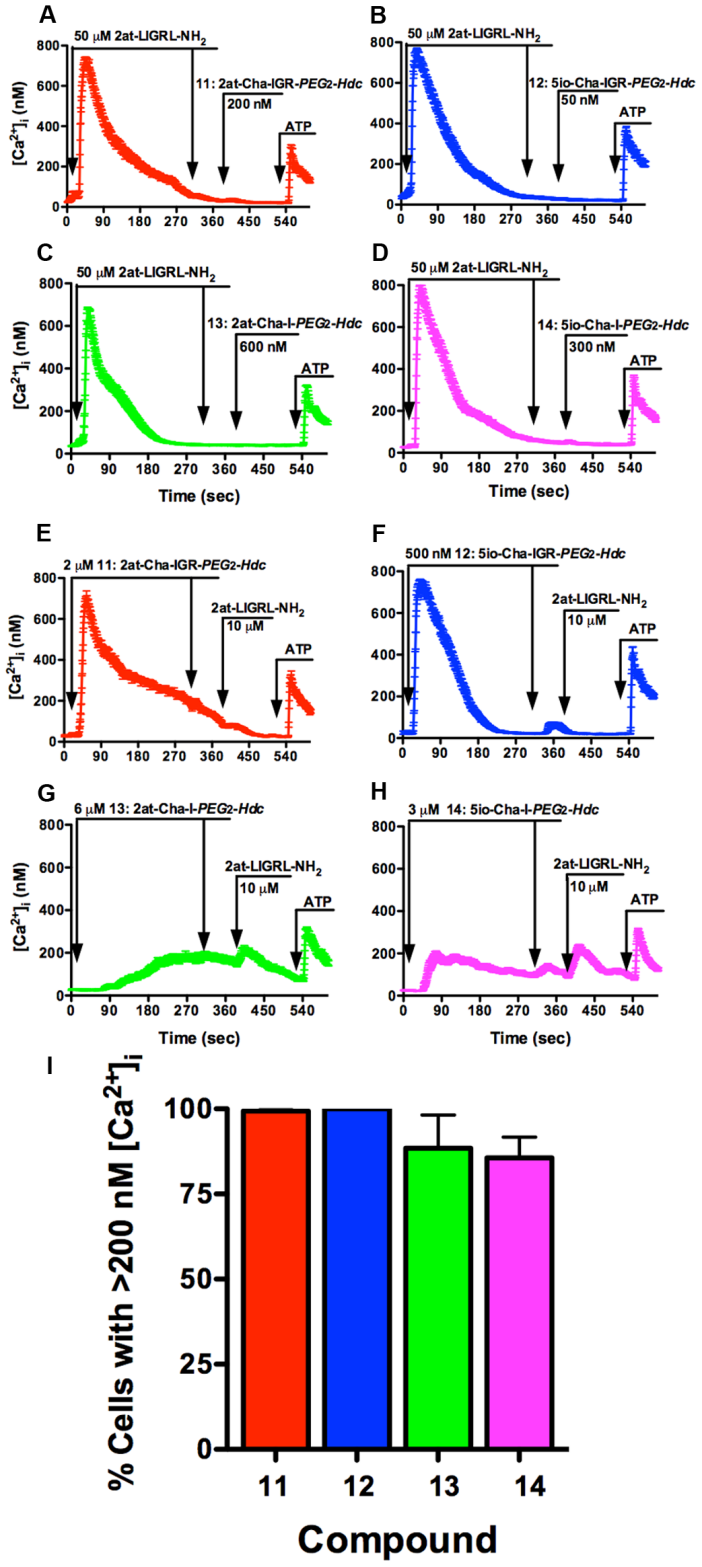
Ca^2+^ desensitization responses for STL agonist compounds 11–14. The top four panels (**A–D**) display traces of the average change in [Ca^2+^]_i._ for all cells in the field of view plotted over time (10 min). In each panel, PAR_2_ desensitization with 50 µM 2at-LIGRL-NH_2_ prevented Ca^2+^ signaling by a second application of 2at-LIGRL-NH_2_ and subsequent addition of PAR_2_ STL agonists — Compound **11**: 2at-Cha-IGR-*PEG*_2_-*Hdc*, 200 nM; **12**: 5io-Cha-LIG-*PEG*_2_-*Hdc*, 50 nM; **13**: 2at-Cha-I-*PEG*_2_-*Hdc*, 600 nM; **14**: 5io-Cha-I-*PEG*_2_-*Hdc*, 300 nM were monitored. Subsequent application of 5 µM ATP in each experiment demonstrated that Ca^2+^ response was intact, and only PAR_2_ dependent pathways were desensitized. In the bottom four panels (**E–H**) 16HBE14o- cells were desensitized with the STL compounds **1–4** at 10 fold their full activation concentrations — Compound **11**: 2at-Cha-IGR-*PEG*_2_-*Hdc*, 2 µM; **12**: 5io-Cha-LIG-*PEG*_2_-*Hdc*, 500 nM; **13**: 2at-Cha-I-*PEG*_2_-*Hdc*, 6 µM; **14**: 5io-Cha-I-*PEG*_2_-*Hdc*, 3 µM. Although compounds **11** and **12** were effective in desensitizing 16HBE14o- cells to 10 µM 2at-LIGRL-NH_2_, desensitization by compounds **13** and **14** was incomplete. In each case, responses to 5 µM ATP remained fully intact. (**I**) Further examination of Ca^2+^ responses in 16HBE14o- cells demonstrated an incomplete Ca^2+^ activation for compounds **13** and **14** persisted at the high agonist concentrations used to desensitize the cells. These data support PAR_2_ specificity for each compound, however the heterocycle-dipeptide STLs do not support full agonistic responses.

Compounds **11–14** were subjected to Ca^2+^ desensitization assays to test for specificity of response. In desensitization assays using 50 µM 2-at-LIGRL-NH_2_ as the specific PAR_2_ ligand to desensitize 16HBE14o- cells, none of compounds **11–14** induced significant Ca^2+^ signaling (**Figure 13A–D**), consistent with PAR_2_ specificity for each of these compounds. Also as above, compounds **11–14** were used at 10x maximal Ca^2+^ signaling response concentrations to assay their ability to desensitization PAR_2_ responses in 16HBE14o- cells to 10 µM 2at-LIGRL-NH_2_. Although compounds **11** and **12** were able to desensitize Ca^2+^ responses in these assays, compounds **13** and **14** did not completely desensitize Ca^2+^ responses to 10 µM 2at-LIGRL-NH_2_ (**Figure 13E–H**). Examination of average Ca^2+^ responses following application of high concentrations of compounds **13** and **14** suggested only partial activation of the 16HBE14o- cells (**Figure 13I**).

## Discussion

We have used a high sensitivity *in vitro* physiological assay combined with synthetic tethered-ligand (STL) approach to evaluate distinct protease-activated receptor-2 (PAR_2_) ligand structure activity relationships (SAR). First, using the RTCA physiological assay, we were able to present a minimal peptide sequence required for full and partial PAR_2_ activation and fully characterize EC_50_s for these truncated compounds. The use of this minimal peptide sequence both *in vitro* and *in vivo* opens new avenues for drug discovery and probing of physiological function at this receptor. Second, we were able to optimize SAR for PAR_2_ ligands with differing, high activity heterocycle (5-isoxazol and 2-aminothiazoyl) substitution of Ser_1_, paired with amino acid sequences naturally occurring in PAR_2_ or the previously used cyclohexylalanine (Cha) substitution for Leu_2_. Such discovery, which is facilitated by the STL approach, is ideal to evaluate otherwise minimally potent and/or questionably selective compounds for PAR_2_ and thus, provide a solid backbone for drug discovery. Finally, we provide detail on PAR_2_ specificity of these compounds, an important point considering the recently discovered pharmacological similarity between PAR_2_ and MrgprC11.

We first evaluated minimal peptide sequence analysis using successive truncation of a known activating peptidomimetic linked to a spaced lipid tether (e.g., [28]). Truncated analogs of 2at-LIGRL-*PEG*_3_-*Hdc* exhibited a descending trend of PAR_2_ activation with a minimal cut off at compound **4**, 2at-LI-*PEG*_3_-*Hdc*. The heterocycle-dipeptide-STL maintained *in vitro* concentration responses (EC_50_ = 310 nM, 95% CI: 262–360 nM) equipotent with the commonly used heterocycle-pentapeptide and heterocycle-hexapeptide (e.g., 2at-LIGRL-NH_2_, RTCA EC_50_ = 310 nM, 95% CI: 240–400 nM; 2-furoyl-LIGRLO-NH_2_, RTCA EC_50_ = 240 nM, 95% CI: 190–290 nM; 6-aminonicotinyl-LIGRL-NH_2_, RTCA EC_50_ = 430 nM, 95% CI: 350–530 nM; [19]).

An alternative approach to assaying minimal peptide structures is the use of single Alanine substitutions (Ala-scan) in SLIGRL-NH_2_ and Ca^2+^ activation assays to assess PAR_2_ activation [15], [16], [34]. Collectively, the Ala-scan studies revealed the importance of Ser_1_ and Leu_2_ for peptide-induced activation of PAR_2_ with full loss of activity when the Leu_2_ was substituted with Ala across all assays. The effects of Ser_1_ substitution with Ala, however, was dependent on the cellular assay with one group demonstrating near complete loss of activity in PAR_2_ expressing kNRK cells [34], another showing significant shift in activity in PAR_2_ expressing oocytes [15], and the third demonstrating only a slight loss of activity in transfected mouse embryonic fibroblasts [16]. Other substitutions were again consistent across assays, where Ala substitutions of Ile_3_ and Arg_5_ decreased PAR_2_ activation while substitutions at Gly_4_ and Leu_6_ did not appreciably alter potency. When multiple Ala substitutions were made to activating peptides, it was shown that SLAAAA-NH_2_ could not activate Ca^2+^ signaling in kNRK cells [34]. Through our STL-truncation approach coupled with the sensitive, *in vitro* physiological responses of RTCA, we found minimal changes in PAR_2_ activation between the parent compound **1** (2at-LIGRL-*PEG*_3_-*Hdc*) when Leu_6_ was removed (compound **2**), successive reductions in potency following removal of Arg_5_-Leu_6_ (**3**) and Ile_4_-Arg_5_-Leu_6_ (**4**) and a complete loss of potency following removal of Ile_3_-Gly_4_-Arg_5_-Leu_6_ (**5**) or Leu_2_-Ile_3_-Gly_4_-Arg_5_-Leu_6_ (**6**). The minimal activating sequence both *in vitro* and *in vivo* required Leu_2_ and Ile_3_ in addition to the heterocycle substitute for Ser_1_ (compound **4**, 2at-LI-*PEG*_3_-*Hdc*). Interestingly, when Ala substitutions were introduced into the receptor and activity uncovered by trypsin activation, the naturally tethered SLAAAA sequence was sufficient for PAR_2_ activation, albeit a less than full cellular response [34]. This minimal activation could not be duplicated using the STL approach, where compound **5** (2at-L-*PEG*_3_-*Hdc*), was inactive both *in vitro* and *in vivo*. It is possible that loss of activity in **5**, could be caused by absence of a peptide backbone or a lost interaction with the side chain of Ile_3_ that may be required in the absence of trypsin cleavage of the receptor. The importance of a peptide backbone is apparent when comparing RTCA activity from compounds **3** (2at-LIG-*PEG*_3_-*Hdc*) and **4** (2at-LI-*PEG*_3_-*Hdc*). The relatively high potency of compound **3** (EC_50_ = 46 nM) was achieved by retention of the Gly_3_, amino acid without any side chain. Compound **4**, however, displayed significantly reduced potency in addition to a delay in time to peak and a reduction in peak Normalized Cell Index. Subsequent reductions in the ability for compound **4** to fully activate Ca^2+^ signaling suggest that activation by this minimal sequence results in only partial agonism of PAR_2_.

PAR_2_ activation is traditionally monitored by Ca^2+^ response following Gq activation and subsequent Ca^2+^ responses (e.g., [18], [19], [20], [21], [28]). However, it is well accepted that activation of PAR_2_ by native proteases or peptidomimetics can result in the recruitment of a variety of G-Proteins and multiple signaling pathways [1], [2]. The RTCA approach used herein to screen PAR_2_ agonists relies on the cellular physiological response that is resultant of the various signaling pathways activated by the candidate drug [30]. Response patterns to individual compounds are reflective of the signaling pathways activated and as such, have been used to classify GPCR ligands into subgroups [35]. Compounds **1–4** tested in these studies displayed RTCA responses consistent with PAR_2_ drugs that elicit both Ca^2+^ and MAPK signaling [19], [28], and do not appear to invoke “biased signaling” via PAR_2_ [4], [25], [26], [36]. Comparison of RTCA responses from primary cultured mouse tracheal epithelial (MTE) cells obtained from wild type or PAR_2_^-/-^ mice successfully demonstrated the need for PAR_2_ expression to invoke physiological responses to these compounds. Traditional “desensitization” studies using Ca^2+^ signaling responses confirmed PAR_2_ specificity of truncated analogues. Extension of the traditional desensitization studies using high concentrations of the newly designed STLs as the agent to desensitize PAR_2_ to a known specific peptidomimetic agonist, 2at-LIGRL-NH_2_ allowed for further understanding of compound/PAR_2_ SAR. For example, the inability of compound **4** to fully desensitize cells at these heightened concentrations is in agreement with the RTCA results that suggest partial agonism by this selective PAR_2_ agonist.

The prototypical peptide activator for PAR_2_, SLIGRL-NH_2_, has recently been shown to contribute to the itch response via an alternative GPCR known to be expressed selectively in sensory neurons, MrgprC11 [12], [37]. Although this receptor is not expressed in 16HBE14o- or MTE cells, and thus not a contributor to the *in vitro* results, activation of this GPCR in *in vivo* experiments could profoundly affect specificity in pain/itch pathways. Application of the parent STL (compound **1**, 2at-LIGRL-*PEG*_3_-*Hdc*) to MrgprC11 transfected CHO cells resulted in a robust Ca^2+^ response, however, this required > 5,000-fold the RTCA EC_50_ concentration. Compound **2**, with a truncated Leu_6_ resulted in limited activity at MrgprC11 at 10 µM and no activity at 1 µM. From these experiments we conclude that retention of the Arg_5_-Leu_6_ is preferred for MrgprC11 activation by our STL compounds. Concentrations required to activate Ca^2+^ responses in transfected MrgprC11 cells demonstrate at least several hundred fold selectivity for PAR_2_ over MrgprC11 by the STL compounds. Finally, the lack of response by 2at-LIGRL-NH_2_ at the EC_50_ concentration for SLIGRL-NH_2_ suggests that the Ser_1_ substitution confers selectivity for PAR_2_ over MrgprC11 in the absence of tethering. Therefore, the approach taken herein has identified highly potent and selective compounds that can be utilized to selectively probe the function of PAR_2_ in sensory biology.

A previous study reported on PAR_2_ activation using peptidomimetic derivatives of the first three amino acids of the natural tethered ligand for PAR_2_ (e.g., Ser_1_-Leu_2_-Ile_3_-NH_2_) at relatively high concentrations (50 µM) and demonstrated partial PAR_2_ activation using Ca^2+^ signaling assays in HEK293 cells [21]. Significantly, one compound from this group, 5io-Cha-Ile-NH_2_ (published as compound **9** in [21] and compound **10** in this report) had an estimated EC_50_ similar to the peptide activator SLIGRLI-NH_2_ [21]. However, the authors noted that lack of solubility of 5io-Cha-I-NH_2_ at high concentrations (100 µM) prevented full EC_50_ determination in their assay. Based on the minimal differences in potency observed in compounds **1** and **2** above, we took advantage of the sensitivity of the RTCA and STL approach to better evaluate EC_50_s of the heterocycle-dipeptides along with longer heterocycle-tetrapeptides. This new group included Ser_1_ substitute heterocycles 2-aminothiazoyl and 5-isoxazol with the Leu_2_ substitute cyclohexylalanine (Cha) and in combination with Ile_3_ or Ile_3_-Gly_4_-Arg_5_ terminated with an amino group. We found that compounds **7–10** all elicited RTCA responses in 16HBE14o- cells, however, only compound **8** (5io-Cha-IGR-NH_2_) elicited a traditional rapid and robust RTCA response typical of full and specific PAR_2_ agonists (e.g., compounds **1–3** herein; [19], [28]). Although not as potent as compounds **1** and **2** above, heterocycle-tetrapeptide STLs were highly potent activators of 16HBE14o- cells, with RTCA EC_50_s of 16 nM (**11**) and 6.8 nM (**12**), and significantly more potent than their corresponding heterocycle-dipeptide STLs (**13**: EC_50_ = 86 nM; **14**: EC_50_ = 43 nM). Direct comparison of 5-isoxazoyl heterocycle with 2-aminothiazoyl heterocycle substitutions resulted in an ∼2 fold decrease in RTCA EC_50_s in both the heterocycle-tetrapeptide and heterocycle-dipeptide STLs. Substitution of Leu_2_ with Cha reduced potency in the heterocycle-tetrapeptides (compare compounds **2** and **11**), whereas the same substitution increased potency in the heterocycle-dipeptide construct (compare compounds **4** and **13**). Although these latter comparisons are tempered by differences in *PEG*_2_ (compounds **9–12**) vs. *PEG*_3_ spacers (compounds **1–4**), such spacer differences using 2at-LIGRL- and 2at-LIGRLO- as parent groups in STLs did not alter potency across assays [28], and thus, the different *PEG* spacers likely do not alter these conclusions. Ca^2+^ desensitization assays using 16HBE14o- cells confirmed specificity of the compounds **11–14** for PAR_2_. However, high concentrations of compounds **13** and **14** could not fully activate Ca^2+^ response nor were they effective at desensitizing 16HBE14o- cells from activation by 10 µM 2-at-LIGRL-NH_2_. These data suggest that the heterocycle-tetrapeptide STLs fully and specifically activate PAR_2_, whereas the heterocycle-dipeptide STLs are PAR_2_ specific, yet partial agonists.

The use of lipid tethering combined with RTCA and supplemented with traditional Ca^2+^ signaling analysis allowed for more robust and interpretable SAR for PAR_2_, including smaller structural nuances that provide an efficient vehicle for future drug development. A strength of this sensitive, tethered ligand approach is the ability to test peptidomimetic ligands in a form that better mimics the natural activation of protease-activated receptors that results in a significant increase in potency. For example, RTCA allowed for separation of potency of peptidomimetic compounds (e.g., 4.5 fold differences in RTCA EC_50_ ranging from 240 nM to 1.1 µM among compounds **7–10**). The increased potency of STL derivatives also allowed for accurate Ca^2+^ signaling studies and confirmation of partial agonism without non-specific effects associated with using high concentrations of newly developed untethered ligands that can obscure SAR. It is interesting that the partial RTCA and Ca^2+^ signaling agonist compound **4** (2at-LI-*PEG*_3_-Hdc) gave a similar response to the full agonists compounds in our *in vivo* assays. These data provide evidence that full agonists (or by analogy, full antagonists) to PAR_2_ may not be needed for full effects *in vivo*. It is accepted that our STL approach increases hydrophobicity in the ligand. Although increased hydrophobicity has traditionally been considered as a negative for building drugs, more recently lipidation of peptides has been recognized as a viable avenue for drug discovery [27]. In closing, we propose that the STL approach will continue to lead to the discovery of high potency peptidomimetics and small molecules as this technique can better identify contrasts between compounds and the resulting higher quality SAR will be enriched with otherwise undetectable structures which may contribute to high fidelity design.
